# Oxytocin affects spontaneous neural oscillations in trauma-exposed war veterans

**DOI:** 10.3389/fnbeh.2015.00165

**Published:** 2015-06-29

**Authors:** Moranne Eidelman-Rothman, Abraham Goldstein, Jonathan Levy, Omri Weisman, Inna Schneiderman, David Mankuta, Orna Zagoory-Sharon, Ruth Feldman

**Affiliations:** ^1^The Gonda Multidisciplinary Brain Research Center, Bar-Ilan UniversityRamat-Gan, Israel; ^2^Department of Psychology, Bar-Ilan UniversityRamat-Gan, Israel; ^3^Department of Obstetrics and Gynecology, Hadassah, Hebrew UniversityRamat-Gan, Israel

**Keywords:** oxytocin, MEG, alpha oscillations, dorsolateral prefrontal cortex (dlPFC), PTSD, veterans

## Abstract

Exposure to combat-related trauma often leads to lifetime functional impairments. Previous research demonstrated the effects of oxytocin (OT) administration on brain regions implicated in post-traumatic stress disorder (PTSD); yet OT’s effects on brain patterns in trauma-exposed veterans have not been studied. In the current study the effects of OT on spontaneous brain oscillatory activity were measured in 43 veterans using magnetoencephalography (MEG): 28 veterans who were exposed to a combat-related trauma and 15 trauma-unexposed controls. Participants participated in two experimental sessions and were administered OT or placebo (PBO) in a double-blind, placebo-control, within-subject design. Following OT/PBO administration, participants underwent a whole-head MEG scan. Plasma and salivary OT levels were assessed each session. Spontaneous brain activity measured during a 2-min resting period was subjected to source-localization analysis. Trauma-exposed veterans showed higher resting-state alpha (8–13 Hz) activity compared to controls in the left dorsolateral prefrontal cortex (dlPFC), specifically in the superior frontal gyrus (SFG) and the middle frontal gyrus (MFG), indicating decreased neural activity in these regions. The higher alpha activity was “normalized” following OT administration and under OT, group differences were no longer found. Increased resting-state alpha was associated with lower baseline plasma OT, reduced salivary OT reactivity, and more re-experiencing symptoms. These findings demonstrate effects of OT on resting-state brain functioning in prefrontal regions subserving working memory and cognitive control, which are disrupted in PTSD. Results raise the possibility that OT, traditionally studied in social contexts, may also enhance performance in cognitive tasks associated with working memory and cognitive control following trauma exposure.

## Introduction

Combat-related post-traumatic stress disorder (PTSD) is a debilitating disorder that often leads to lifetime impairments in cognitive processing, social relationships, and emotion regulation (Yehuda, 2002; American Psychiatric Association, 2013). Prevalence studies have shown that between 2 and 17% of US veterans develop PTSD (Richardson et al., 2010), particularly those who witnessed injury or death of another person (Smith et al., 2008). Furthermore, many veterans show substantial functional impairment, work and family problems, and dramatic reduction in quality of life even decades after combat (Schnurr et al., 2009). These are also observed in veterans who do not meet full criteria for PTSD (Schnurr et al., 2000; Grubaugh et al., 2005; Jakupcak et al., 2007).

Research has shown significant alterations in brain structure and function in cases of PTSD in a number of regions, including the amygdala and the prefrontal cortex (PFC; Karl et al., 2006; Hayes et al., 2012; Pitman et al., 2012). Functional neuroimaging studies using different paradigms, such as symptom provocation or cognitive and emotional tasks, have demonstrated hyper-activation of the amygdala (Liberzon et al., 1999; Rauch et al., 2000; Bryant et al., 2005; Fonzo et al., 2010; Simmons et al., 2011) and hypo-activation of the PFC (Shin et al., 2001; Lanius et al., 2003; Williams et al., 2006; Milad et al., 2009), and some have shown inversed relation of activity (Shin et al., 2004, 2005) and abnormal connectivity (Stevens et al., 2013) between the amygdala and the PFC. Comparable findings were also observed during a resting-state, i.e., in the absence of task demands (Chung et al., 2006; Sripada et al., 2012; Yan et al., 2013). It has been suggested that amygdala hyper-responsivity and impaired top-down prefrontal control mediate the dysregulation of fear and threat-related processing in PTSD (Rauch et al., 2006). More recently it has been proposed (Patel et al., 2012) that abnormalities in three different neuro-cognitive networks may explain the range of symptoms seen in PTSD. One of these networks, the central executive network (Menon, 2011), is associated with high-level cognitive functions, such as decision making, attention control, and working memory, and is anchored in the dorsolateral prefrontal cortex (dlPFC), a region critical to proper cognitive control over emotions (Ochsner et al., 2002; Phan et al., 2005). Thus, processes typical of PTSD such as negative appraisals of stimuli, as well as the disrupted cognitive functioning seen in PTSD, may be related to aberrant top-down signaling from this network (Patel et al., 2012).

Decreased activity in the dlPFC has been observed in PTSD during the performance of cognitive tasks (Clark et al., 2003; Falconer et al., 2008; Rabinak et al., 2014) and during a resting-state (Yan et al., 2013) and such decrease is associated with PTSD symptom severity (Falconer et al., 2008; Yan et al., 2013). Studies assessing the effects of therapy on brain function in PTSD have shown that positive effects were associated with activation in frontal regions, including the dlPFC (Cohen et al., 2004; Farrow et al., 2005; Peres et al., 2007; Lindauer et al., 2008; Boggio et al., 2010; Watts et al., 2012; Thomaes et al., 2014). These studies suggest that the dlPFC may be a target region for intervention effort in PTSD and that improved functioning in this region may serve as an index of positive therapeutic effects.

Intranasal administration of oxytocin (OT), a nine-amino-acid neuropeptide implicated in mammalian caregiving and sociality (Feldman, 2012; Carter, 2014), has been shown to exert regulatory effects on brain patterns in healthy individuals exposed to threatening stimuli and in individuals with a variety of psychiatric conditions due to its anxiolytic properties (Meyer-Lindenberg et al., 2011; Bethlehem et al., 2013; Weisman and Feldman, 2013; MacDonald and Feifel, 2014). The anxiolytic and stress protective effects of OT have been demonstrated in animal (Windle et al., 2004; Blume et al., 2008; Viviani et al., 2011; Peters et al., 2014) and human (Heinrichs et al., 2003; Light et al., 2005; Gordon et al., 2008; Ditzen et al., 2009) research. In animals, research has shown that the induction of endogenous OT release from the central amygdala decreases fear responses (Knobloch et al., 2012). In humans, anxiolytic effects have been demonstrated by the attenuation of amygdala activation to fearful stimuli following OT administration, as observed in functional MRI studies with healthy adults (Kirsch et al., 2005; Domes et al., 2007). In addition, OT has been shown to increase resting-state functional connectivity between the amygdala and frontal regions that are critical for fear extinction and regulation of emotions (Sripada et al., 2013), further supporting its anti-stress effects. The anxiolytic and stress reducing properties of OT have been suggested as one mechanism that underpins the well-established pro-social effects of OT in humans (Churchland and Winkielman, 2012; MacDonald and Feifel, 2013).

Studies utilizing intranasal OT administration in clinical populations have shown that OT improved core symptoms and aberrant brain patterns in cases of schizophrenia (Feifel et al., 2010), autism (Domes et al., 2013; Gordon et al., 2013), and social anxiety (Labuschagne et al., 2010; Dodhia et al., 2014). Studies have also shown that OT administration improved symptom severity (Yatzkar and Klein, 2010) and reduced the physiological response to combat imagery (Pitman et al., 1993) in PTSD. The effects of OT administration on reducing the amygdala fear-response (Kirsch et al., 2005; Domes et al., 2007) and on increasing amygdala-prefrontal connectivity in healthy adults (Sripada et al., 2013) and in adults with generalized social anxiety disorder (Dodhia et al., 2014), suggest that OT may exert a regulatory function on brain patterns following exposure to combat-related trauma.

In light of the above, the current study examined the effects of OT administration on spontaneous (resting-state) brain activity in trauma-exposed war veterans. Spontaneous activity has been used to examine the intrinsic functional activity of the brain (Raichle and Mintun, 2006) and has been shown to index risk for various psychiatric disorders including PTSD (Buckner and Vincent, 2007). In PTSD, abnormal oscillatory activity in frontal regions and in the insula was found during resting-state in trauma-exposed individuals using magnetoencephalography (MEG; Kolassa et al., 2007). In another MEG study, resting-state activity differentiated patients from controls, suggesting that spontaneous brain patterns may serve as neuromarkers for the disorder (Georgopoulos et al., 2010). Brain oscillations reflect synchronized activity of large populations of neurons and support normal brain function (Buzsáki and Draguhn, 2004). In the current study, we were particularly interested in alpha rhythm, the predominant oscillatory frequency in humans during eyes-closed rest (Nunez et al., 2001). Alpha activity has been associated with decreased cortical activity (Goldman et al., 2002; Laufs et al., 2003a; Jensen and Mazaheri, 2010), and anomalies in resting-state alpha have been observed in various psychiatric disorders (Ciesielski et al., 2007; Hinkley et al., 2011; Cornew et al., 2012), including combat-related PTSD (Jokić-begić and Begić, 2003; Huang et al., 2014).

We further measured whether post-traumatic symptoms and peripheral OT levels may be associated with the effects of OT administration on spontaneous oscillatory brain patterns.

## Materials and Methods

### Participants

At the first stage of the study, 190 young male veterans (age < 35 years) were recruited. These included 158 veterans who served in the Israel Defense Force (IDF) in active combat units during the past 8 years, participated in active battle that involved life endangerment, and witnessed injury or death of a comrade. Thirty-two veterans matched for age and education, who served in the IDF during the same period in non-combat units (e.g., intelligence, technical support) and were not exposed to active combat were recruited as controls. All veterans completed the Post-Traumatic Stress Diagnostic Scale (PDS; Foa et al., 1997) for the assessment of post-traumatic symptom severity and in order to ensure that members of the control group were free of post traumatic symptoms. Twenty-eight veterans from the trauma-exposed group with non-zero PDS scores (mean age = 27 years, SD = 1.81) and 16 controls with 0 PDS scores (mean age = 28.94 years, SD = 3.02) who met inclusion criteria participated in the OT administration and MEG study, as described below. Most members of the trauma-exposed group experienced the traumatic event 3 or 5 years before the study, during two distinct military operations, and the others in various other operations in the period between 3 to 8 years before the study.

Participants were recruited via advertisement in the community and were matched for demographic status. Subjects were right-handed as measured by the Edinburgh Handedness Questionnaire except for three subjects from the exposed group who were neutral (*N* = 1) or left handed (*N* = 2). Exclusion criteria included serious physical injury, current or past neurological disorders, serious medical problems, and regular use of medication. War-exposed veterans were not exposed to trauma other than combat. Control veterans were not exposed to trauma of any kind and had no present psychiatric disorder. The study was approved by the Institutional Review Board of Bar-Ilan University and after complete description of the study, written informed consent was obtained from all participants.

### Experimental Procedure

#### Clinical Diagnosis

All participants were diagnosed by clinical psychologists blind to trauma history supervised by a psychiatrist using the Structured Clinical Interview for DSM-IV Axis I Disorders (SCID-I; First et al., 1997). Among controls, none received any Axis-I psychiatric diagnosis. Among trauma-exposed veterans 11 were diagnosed with PTSD and five of them showed co-morbidity which included depression (*N* = 3), dysthymia (*N* =‘2), phobia (*N* = 2), panic attacks (*N* = 1), and OCD (*N* =‘2). Trauma-exposed veterans who did not meet criteria for a full PTSD diagnosis showed no difference in their post-traumatic symptom scores in any symptom cluster (re-experience, avoidance, hyper-arousal) or in the functional impairment domain on the PDS. Mean PDS symptom severity scores were 19.72 (SD = 9.12) for trauma-exposed veterans who met DSM-IV criteria for a full PTSD Axis-I diagnosis and 16.13 (SD = 7.22) for trauma-exposed veterans who did not meet criteria for full PTSD diagnosis (*F*_(1,25)_ = 1.25, NS). We therefore combined the two groups into a single group. This is consistent with the DSM-V approach, which advocates a dimensional approach to psychiatric disorders as indexed by symptom severity profiles (American Psychiatric Association, 2013).

#### Experimental Design

The study employed a double-blind, placebo (PBO)-control, within-subject design. Each subject participated in two similar experimental sessions approximately a week apart (mean = 7.5 days, SD = 2). Baseline (T1) blood and saliva samples were collected in each session. Following, participants self-administered 24 IU of either OT (Syntocinon Spray, Novartis, Switzerland; three puffs per nostril, each containing 4 IU) or PBO. PBO was custom-designed by commercial compounding pharmacy to match drug minus the active ingredient. Administration order was counterbalanced, and participants and experimenters were blind to drug condition. Experimental sessions were conducted between 14:30 and 16:00 pm to accommodate diurnal variations in OT.

Forty-five minutes after OT/PBO administration, spontaneous brain activity was measured using MEG during a 2 min period when subjects rested with their eyes closed. Two additional saliva samples were collected in each experimental session, one before (T2) and one after (T3) the MEG scanning.

#### Brain Activity Recording and Analysis

MEG recordings were conducted with a whole-head, 248-channel magnetometer array (4-D Neuroimaging, Magnes 3600 WH) in a magnetically-shielded room with a sample rate of 1017 Hz and online 1–400 Hz band-pass filter in a supine position. External noise (e.g., power-line, mechanical vibrations) and heartbeat artifacts were removed from the data as previously described (Tal and Abeles, 2013). Signal pre-processing at the sensor level was carried out using MATLAB and the FieldTrip toolbox (Oostenveld et al., 2011). The data were segmented into 1000 ms epochs, filtered in the 1–80 Hz range with 10 s padding and were baseline-corrected. The data epochs were visually inspected for eye movement and muscle artifacts which were excluded from further analysis.

For source estimation, a template MRI (Collin27) was modified to fit each subject’s digitized head shape using SPM8 (Wellcome Department of Imaging Neuroscience, University College London, London, UK).^1^ Synthetic aperture magnetometry (SAM) beamformer (Robinson and Vrba, 1999) was applied with a spatial resolution of 0.5 cm for each experimental condition (OT or PBO) at four frequency bands: delta (1–4 Hz), theta (4–8 Hz), alpha (8–13 Hz) and beta (13–20 Hz). Individual source power maps were reconstructed using pseudo-Z statistic. Following transformation into a common coordinate space (Talairach) clusters with significant effects were identified by a voxel-level *t*-test and were corrected for multiple comparisons based on a Monte Carlo simulation of random noise distribution (Forman et al., 1995). The results were superimposed on the MRI template as statistical maps, showing regions of statistically significant difference in activity. The voxel average activity in clusters with significant group differences at baseline (under PBO) was used in the following analyses.

#### Oxytocin Collection and Determination

Blood was drawn at baseline, before OT/PBO administration, during each visit from antecubital veins into nine milliliter chilled vacutainer tubes containing lithium heparin supplemented with 400 KIU of Trasylol (Trasylol–Bayer, Germany) per one milliliter blood. Blood samples were kept ice-chilled for up to 2 h before centrifuged at 4°C at 1000 × g for 15 min. Supernatants were collected and stored at −80°C until assayed.

Saliva samples were collected three times each visit as described above. OT from saliva was collected by Salivate (Sarstedt, Rommelsdorft, Germany). Salivates were kept ice-chilled for up to 1 h before centrifuged at 4°C at 1500 × g for 15 min. Liquid samples were stored at −80°C. To concentrate the samples by 3–4 times, liquid samples were lyophilized over-night and kept in −20°C until assayed. Dry samples were reconstructed in the assay buffer immediately before analysis by OT EIA commercial Kit, consistent with previous research (Feldman et al., 2010).

Determination of OT was performed using commercial ELISA kit (Assay Design, MI, USA; through ENZO, NY, USA), consistent with our previous research (Feldman et al., 2011; Weisman et al., 2013). Measurements were performed in duplicate and concentration of samples calculated using MATLAB according to relevant standard curves. The intra-assay and inter-assay coefficients were <12.3 and <14.5%, respectively.

Immunoassay methods (EIA/ELISA) for the quantitative determination of OT and is considered the gold standard in current OT research and has shown to be sensitive and reliable (Kramer et al., 2004), and unlike the earlier radioimmunoassay (RIA) method it does not involve the use of radioactive substances (but note several methodological issues, see “Limitations” Section).

Two measures of peripheral OT were computed:

*Baseline plasma OT* was the average of baseline (T1) OT levels in the two visits, which were highly correlated, (*r* = 0.97, *p* < 0.0001).
*Salivary OT reactivity* was measured as the increase in OT from baseline (T1) after OT administration (T2 + T3 2 minus T1 in the OT session).

## Results

### Group Differences in Spontaneous Brain Activity Under Placebo

**Figure 1:**
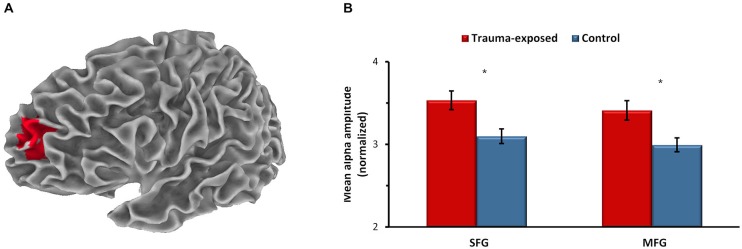
**Between-group differences in spontaneous brain activity at baseline (placebo). (A)** Dorsolateral prefrontal cluster of the left superior frontal gyrus (SFG) and the left middle frontal gyrus (MFG) showing higher alpha activity in the trauma-exposed group compared to the control group, *p* < 0.05, corrected. *x*, *y*, *z* of maximal voxels in Montreal Neurological Institute (MNI) coordinates = −17, 59, 22 and −27, 64, 27, for SFG and MFG, respectively. Averaged alpha activity over all voxels in the SFG and in the MFG **(B)**. *p < 0.05. Error bars represent SEM.

Analysis was first performed for the PBO condition in order to assess group differences in spontaneous brain activity at baseline. Group differences were found only in alpha-band activity (8–13 Hz), located in a cluster of two regions within the left dlPFC (Figure 1A): the left superior frontal gyrus (SFG) and the left middle frontal gyrus (MFG). In these regions, the trauma-exposed group showed increased alpha activity compared to the control group (Figure 1B; *F*_(1,40)_ = 6.88, *p* = 0.014, and *F*_(1,40)_ = 5.82, *p* = 0.021, for SFG and MFG, respectively).

### Effects of Oxytocin on Spontaneous Brain Activity

**Figure 2:**
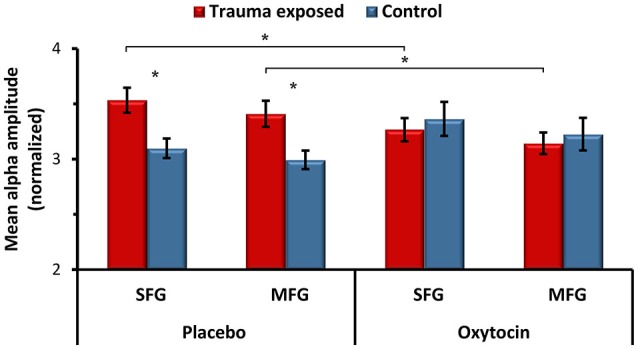
**The effects of oxytocin (OT) administration on spontaneous alpha activity.** Averaged alpha activity over all voxels in the left SFG and in the left MFG in the placebo and OT conditions. *p < 0.05. Error bars represent SEM.

In the next stage, we tested the effect of OT administration on alpha activity in the two dlPFC regions that showed group differences at PBO, the left SFG and the left MFG. A repeated measure analysis of variance (ANOVA) showed a significant interaction effect of group and OT administration for both the SFG (*F*_(1,38)_ = 5.76, *p* = 0.021), and the MFG (*F*_(1,38)_ = 4.89, *p* = 0.033), indicating that OT’s effect differed by group (Figure 2). Specifically, OT administration significantly decreased the heightened alpha activity among trauma-exposed veterans, and under OT, group differences were no longer found. In the control group however, OT increased the alpha activity, but this finding was not statistically significant.

### Peripheral Oxytocin

No differences were found between groups in plasma and salivary OT levels at baseline or in OT reactivity.

### Spontaneous Alpha Activity, Peripheral Oxytocin and Symptom Severity

Alpha activity at baseline (at PBO) correlated with peripheral measures of OT for the entire sample and with symptom severity in the trauma-exposed group. Higher alpha in the left SFG was associated with lower levels of baseline plasma OT (*r* = −0.38, *p* = 0.014), and higher alpha in the left MFG was associated with lower salivary OT reactivity (*r* = −0.35, *p* = 0.025). Interestingly, more re-experiencing symptoms correlated with higher baseline alpha in both the left SFG, (*r* = 0.43, *p* = 0.027), and the left MFG, (*r* = 0.39, *p* = 0.045). Re-experiencing symptoms index the uncontrolled re-living of the trauma in the form of thoughts and images of the traumatic event, nightmares, flashbacks, psychological distress and physiological reactivity to trauma-reminders.

## Discussion

Results of the current study, the first to test the brain response of trauma-exposed veterans to OT administration, demonstrate that OT significantly attenuated the aberrant alpha activity in dorsolateral prefrontal regions implicated in working memory and cognitive control, in trauma-exposed veterans. Spontaneous oscillations in the alpha range are the predominant electrophysiological signal in healthy adults during rest when the attention system is not recruited toward a specific task (Nunez et al., 2001). Alpha oscillations are associated with decreased cortical activity, are thought to reflect functional inhibition (Jensen and Mazaheri, 2010), and are negatively correlated with the strength of BOLD fMRI signal in frontal and parietal cortical areas that support attention and related cognitive processes (Laufs et al., 2003b). This suggests that higher alpha power provides an index of decreased cortical activity in these regions, and our findings suggest that OT may have some impact, albeit momentarily, on prefrontal structures suggested as among those underpinning the functional impairment in PTSD (Patel et al., 2012).

Specifically, we found that OT attenuated the increased alpha power observed among trauma-exposed veterans in two frontal regions, the left SFG and the left MFG. Neuroimaging and human lesion studies indicated that both the SFG and MFG are critical for working memory and cognitive control (Aron et al., 2004; Egner and Hirsch, 2005; du Boisgueheneuc et al., 2006; Jurado and Rosselli, 2007). In patients with left SFG lesion, impairments to working memory increased as the task demands became more complex (du Boisgueheneuc et al., 2006) and in patients with left MFG lesion (Aron et al., 2004) the degree of cognitive control impairments correlated with the extent of damage to this region. Studies have repeatedly shown dysfunction in working memory (Elzinga and Bremner, 2002), attention, and cognitive control (Banich et al., 2009) in patients with PTSD, including combat-related PTSD (Vasterling et al., 1998). Our findings, therefore, raise the possibility that OT, which has been studied almost entirely in the social context, may also act to enhance performance in cognitive tasks associated with working memory and cognitive control which are disrupted in PTSD or may be used in conjunction with cognitive-behavior therapy, but this hypothesis is only preliminary and requires much further systematic research in larger samples.

The difficulties seen in PTSD patients in working memory, attention, and cognitive control (Banich et al., 2009) have been shown to index PFC disturbances. Neuroimaging studies found hypo-activity of the PFC, hyper-activity of the amygdala (Hayes et al., 2012; Pitman et al., 2012), and inverse relation of activity in the amygdala and the PFC (Shin et al., 2004, 2005) in cases of PTSD. Abnormal activity in the central executive network, a brain network which is situated in the dlPFC and is associated with high-level cognitive functions (Menon, 2011), has been implicated in some of the behavioral difficulties in PTSD (Patel et al., 2012). The increase in activity in the left SFG and MFG following OT administration, indexed by the decrease in alpha activity found here, may suggest that OT exerts anxiolytic effects by enhancing activity in an attention-related network implicated in the application of cognitive, goal-directed selection to sensory information and responses (Egner and Hirsch, 2005). Furthermore, previous studies indicated that the SFG and MFG are sensitive to OT administration in patient populations. Among depressed individuals OT increased activity in the SFG and MFG (Pincus et al., 2010) and among children with autism OT increased activity in the MFG (Gordon et al., 2013). Our findings, therefore, are consistent with the current effort to specify the effects of OT on brain functioning in different psychiatric conditions (Weisman and Feldman, 2013).

Our findings are also in line with research demonstrating structural (Geuze et al., 2008; Tavanti et al., 2012) and functional (Chung et al., 2006; Morey et al., 2008) alterations in the SFG and MFG in patients with PTSD. Moreover, the degree of alterations in these regions was found to correlate with the severity of PTSD symptoms (Morey et al., 2008; Tavanti et al., 2012). Our data similarly show that the degree of SFG and MFG hypo-activity at baseline, indexed by increased alpha activity, correlated with the number of re-experiencing symptoms, including repeated flashbacks, sensory-based memories, and uncontrolled reliving of trauma. A negative correlation between spontaneous activity in the dlPFC and re-experiencing symptoms has been reported in a previous work (Yan et al., 2013) and it has been suggested to be related to the role of this region in inhibiting unwanted memories (Anderson et al., 2004; Yan et al., 2013). Additionally, research has shown that increased activity in SFG and MFG following cognitive behavior therapy was related to PTSD symptom improvement (Lindauer et al., 2008). Finally, evidence indicates that frontal alpha activity is sensitive to therapy in PTSD (Kluetsch et al., 2014). Taken together, these studies underscore the sensitivity of these dorsolateral prefrontal regions to both symptom severity and treatment effects in PTSD. This is in line with perspectives suggesting that the prefrontal deficits in PTSD are not innate but acquired following the onset of the disorder and may thus be more amenable to intervention (Admon et al., 2013).

We are aware of no prior study that examined the effects of OT administration on brain response in trauma-exposed combat veterans and the findings therefore suggest that much further research is warranted to fully test the potential beneficial effects of OT in this context. Our findings that OT administration was effective for some individuals but not for others is consistent with the general observation that OT’s effect is influenced by various personal factors (Bartz et al., 2011b), including the presence of psychiatric symptoms, as demonstrated in a number of studies showing differential effects of OT administration on brain activity among patients and controls (Labuschagne et al., 2010; Pincus et al., 2010; Dodhia et al., 2014). Such data strengthen the view of the complex nature of OT (e.g., Bartz et al., 2011b; Weisman and Feldman, 2013) and support the current call to implement personalized intervention that is guided by the individual’s symptomatology for targeted and effective treatments. Very little research examined the involvement of the OT system in PTSD and much further research is required. The observation that OT’s effects are influenced by personal as well as contextual factors (Bartz et al., 2011b) and importantly, are not always positive (e.g., Bartz et al., 2011a; De Dreu et al., 2011; Shalvi and De Dreu, 2014) emphasize the need to further examine its effects in PTSD, also in other contexts, using different experimental tasks, in addition to the currently used resting-state assessment.

Overall, the high number of returning veterans with traumatic war experiences and the vulnerability of this population to develop PTSD (Kessler et al., 1995; Richardson et al., 2010), underscore the importance and potential clinical utility of these findings, but, at the same time, emphasize the preliminary nature of our study. Much further research is required to describe the effects of OT on oscillatory brain activity at rest and in response to various tasks, to test mechanisms by which OT impacts other neurohormonal systems, such as the HPA, dopamine, or immune systems, and to specify the types of psychological interventions that may work in concert with OT to increase well-being among patients suffering from post-traumatic distress.

## Limitations

Several study limitations should be remembered in the interpretation of the findings. First, although our study is large for a brain study, involving 86 brain scans—among the largest OT-administration brain studies—the number of veterans with PTSD is relatively small and the findings require further validation and specification before generalizability can be made. Second, we specifically chose veterans who are young, relatively functional, and free from medical illness, and further research is required to test whether our findings generalize to veterans with long-lasting PTSD, medical conditions, or functional impairment. Next, some methodological concerns regarding OT measurement should be considered. First, there is still uncertainty as to what other molecules are possibly being measured along with OT using the commonly applied methods for OT levels determination, (i.e., RIA vs. EIA), especially when pre-analysis extraction procedure is not included (McCullough et al., 2013), as was the case in our study. Further validation and standardization are needed to better understand the meaning of these measures. Second, while OT is released by the pituitary gland into the blood stream and is presumed to be the primary source of salivary OT (Carter et al., 2007), the relation between peripheral OT measures and OT’s function within the CNS is still not well understood; therefore, the physiological meaning of these measures should be interpreted with caution. Considering plasma vs. salivary OT measures, saliva sampling is advantageous since it is a convenient, non-invasive and less stressful procedure (Carter et al., 2007). However, the plasma measure is considered a more direct one, yet we found in several studies that plasma and saliva measures show medium-level correlations (Feldman et al., 2011). Finally, it is important to complement brain activity in the absence of task (i.e., “resting state”) with response patterns to specific tasks related to fear stimuli in general or to the individual’s specific traumatic memories for a fuller understanding of brain patterns underpinning PTSD and their response to oxytocin administration.

## Conflict of Interest Statement

The authors declare that the research was conducted in the absence of any commercial or financial relationships that could be construed as a potential conflict of interest.

## Footnotes

^1^www.fil.ion.ucl.ac.uk

## Abbreviations

OT: Oxytocin
PBO: placebo
dlPFC: dorsolateral prefrontal cortex
SFG: superior frontal gyrus
MFG: middle frontal gyrus.
